# Raising the Bar of PK/PD Target Attainment of Beta-Lactams in Daily Clinical Practice: An Effective Strategy to Overcome Resistance Development to Novel Beta-Lactams?

**DOI:** 10.3390/idr18010003

**Published:** 2025-12-23

**Authors:** Milo Gatti, Federico Pea

**Affiliations:** 1 Department of Medical and Surgical Sciences, Alma Mater Studiorum University of Bologna, 40138 Bologna, Italy; milo.gatti2@unibo.it; 2 Clinical Pharmacology Unit, Department for Integrated Infectious Risk Management, IRCCS Azienda, Ospedaliero-Universitaria di Bologna, 40138 Bologna, Italy

## 1. Introduction

The increase of infections caused by difficult-to-treat resistant (DTR) Gram-negatives is becoming an ever-growing remarkable issue for public health [1,2,3]. Specifically, carbapenem-resistant *Enterobacterales*, DTR *Pseudomonas aeruginosa*, and carbapenem-resistant *Acinetobacter baumannii* (CRAB) are pathogens considered critical-priority nowadays by the World Health Organization [4], and are responsible for severe infections with high morbidity and mortality rates [5,6].

In this challenging scenario, novel beta-lactams (BL) and BL/beta-lactamase inhibitor combinations (BL/BLIc) are currently recommended by both the ESCMID guidelines and the IDSA guidance as first-line treatment [7,8]. In recent years, agents like cefiderocol, ceftazidime/avibactam, ceftolozane/tazobactam, meropenem/vaborbactam, imipenem/relebactam, and lastly aztreonam/avibactam have allowed significant improvements in clinical outcomes of these settings compared to best available therapies [9,10,11,12,13,14]. Unfortunately, the selective pressure associated with their extensive use has led to resistance development, whose prevalence is worryingly increasing in several centers worldwide [15,16,17,18]. The CACTUS study was a multicenter, retrospective, observational study carried out in 28 US hospitals over 8 years (2016–2023) among 420 eligible patients having *Pseudomonas aeruginosa* pneumonia or bacteremia treated with ceftolozane-tazobactam or ceftazidime–avibactam (210 in each treatment group). Regarding this, it showed that among patients having baseline isolates tested for susceptibility (350/420), resistance developed in more than 20% of cases of both arms (22%, 38/173 with ceftolozane–tazobactam and 23% 40/177 with ceftazidime–avibactam) [14]. This and several other epidemiological studies make the adoption of proper corrective strategies a compelling clinical need for counteracting this trend and for preserving the activity of the novel BL and BL/BLIc against these pathogens over time.

Interestingly, it was recently shown that maximizing the performance of these agents by raising the threshold of the pharmacokinetic/pharmacodynamic (PK/PD) target during their clinical use could be a potentially effective strategy for overcoming this issue [19]. Specifically, several recent studies showed that shifting from the conservative standard PK/PD target of 50–100% time above the MIC (T _> MIC_) to a 4–8-fold higher threshold, namely 100%T_> 4 x MIC_, defined as an aggressive PK/PD target, may represent an innovative frontier for minimizing the resistance development related to their use, especially among critically ill patients [19,20,21,22,23]. It should also be remembered that when dealing with BL/BLIc, the aggressive PK/PD target must involve both the BL and the BLI, namely must be joint [24].

An incipit on this topic came from a recent meta-analysis of 21 observational studies assessing the impact of PK/PD target attainment of traditional beta-lactams on clinical outcome of Gram-negative infections in critically ill patients [21]. It was shown that attaining an aggressive PK/PD target was more protective against resistance development risk compared to a conservative one (OR 0.06; 95% CI 0.01–0.29) [21]. Two main tools may be helpful for maximizing the likelihood of aggressive PK/PD target attainment, namely, delivering the BL by extended or continuous infusion (CI) and optimizing plasma exposure by means of a TDM-guided approach [25]. Regarding the former, a recent meta-analysis of 21 observational studies showed that delivering BL by prolonged and/or CI was the only independent predictor decreasing the risk of aggressive PK/PD target non-attainment. Regarding the latter, a meta-analysis of 11 studies showed that among critically ill patients receiving treatment with BL, adopting a TDM-guided strategy was effective in increasing the rates of both optimal PK/PD target attainment (RR 1.85; 95%CI 1.08–3.16) and microbiological eradication (RR 1.14; 95%CI 1.03–1.27) compared to a standard approach [26]. This may be effective especially whenever the TDM-guided strategy is coupled with expert clinical pharmacological advice of the TDM results [27,28].

Moving to the specific setting of novel BL/BLIc, recent studies showed that implementing antimicrobial stewardship programs focused on both delivering the drug by continuous infusion (CI) and optimizing the likelihood of aggressive PK/PD target attainment by means of a TDM-guided approach may be very effective for this purpose. A pre-post quasi-experimental study assessed the impact of a multidisciplinary approach (involving the infectious disease consultant, the clinical microbiologist, and the clinical pharmacologist) aimed at attaining an aggressive joint PK/PD target of ceftazidime–avibactam on the treatment outcome of KPC *Klebsiella pneumoniae*-related infections and on the prevention of ceftazidime–avibactam resistance development [23]. Overall 228 patients were involved, namely 116 undergoing standard management in the pre-intervention phase and 102 undergoing the multidisciplinary management in the post-intervention phase in which real-time TDM-guided PK/PD target attainment optimization was provided. It is noteworthy that in the post-intervention phase, ceftazidime–avibactam was administered more frequently by CI (96.1% vs. 31.9%; *p* < 0.001) and attaining aggressive joint PK/PD target was protective against both microbiological failure (OR 0.03; 95% CI 0.005–0.20) and 90-day resistance development (OR 0.07; 95% CI 0.01–0.69) [23]. Similar findings were observed in another pre-post quasi-experimental study carried out among 85 patients having *Pseudomonas aeruginosa* BSIs and/or VAP treated with CI ceftolozane–tazobactam monotherapy [29]. Thirty-seven patients receiving a TDM-guided strategy in the post-intervention phase were compared with 48 patients receiving standard management with CI ceftolozane/tazobactam monotherapy in the pre-intervention phase [29]. In the post-intervention phase, all patients attained an aggressive PK/PD target, microbiological eradication rate trended to be higher (75.8% vs. 56.3%; *p* = 0.10), and 30-day resistance development almost halved (10.8% vs. 18.8%; *p* = 0.37) [29].

Overall, these very encouraging findings emerging from proof-of-concepts real-world studies may support the contention that aggressive PK/PD target attainment may be the goal for minimizing the risk of resistance development to novel BL during treatment of DTR Gram-negative infections, thus preserving efficacy over time. Obviously, prospective confirmatory studies involving adequate sample size are warranted. Meanwhile, clinicians are encouraged to adopt two helpful strategies for maximizing the likelihood of aggressive PK/PD target attainment when dealing with severe Gram-negative infections in critically ill patients, namely delivering BL by prolonged or even better by CI and implementing a TDM-guided approach whenever feasible.

## Data Availability

Not applicable.

## References

[1] Murray C.J.L., Ikuta K.S., Sharara F., Swetschinski L., Aguilar G.R., Gray A., Han C., Bisignano C., Rao P., Wool E. (2022). Global Burden of Bacterial Antimicrobial Resistance in 2019: A Systematic Analysis. Lancet.

[2] Naghavi M., Vollset S.E., Ikuta K.S., Swetschinski L.R., Gray A.P., Wool E.E., Aguilar G.R., Mestrovic T., Smith G., Han C. (2024). Global Burden of Bacterial Antimicrobial Resistance 1990–2021: A Systematic Analysis with Forecasts to 2050. Lancet.

[3] Macesic N., Uhlemann A.-C., Peleg A.Y. (2025). Multidrug-Resistant Gram-Negative Bacterial Infections. Lancet.

[4] Tacconelli E., Carrara E., Savoldi A., Harbarth S., Mendelson M., Monnet D.L., Pulcini C., Kahlmeter G., Kluytmans J., Carmeli Y. (2018). Discovery, Research, and Development of New Antibiotics: The WHO Priority List of Antibiotic-Resistant Bacteria and Tuberculosis. Lancet Infect. Dis..

[5] Paniagua-García M., Bravo-Ferrer J.M., Pérez-Galera S., Kostyanev T., de Kraker M.E.A., Feifel J., Palacios-Baena Z.R., Schotsman J., Cantón R., Daikos G.L. (2024). Attributable Mortality of Infections Caused by Carbapenem-Resistant Enterobacterales: Results from a Prospective, Multinational Case-Control-Control Matched Cohorts Study (EURECA). Clin. Microbiol. Infect..

[6] Huh K., Chung D.R., Ha Y.E., Ko J.-H., Kim S.-H., Kim M.-J., Huh H.J., Lee N.Y., Cho S.Y., Kang C.-I. (2020). Impact of Difficult-to-Treat Resistance in Gram-Negative Bacteremia on Mortality: Retrospective Analysis of Nationwide Surveillance Data. Clin. Infect. Dis..

[7] Paul M., Carrara E., Retamar P., Tängdén T., Bitterman R., Bonomo R.A., de Waele J., Daikos G.L., Akova M., Harbarth S. (2022). European Society of Clinical Microbiology and Infectious Diseases (ESCMID) Guidelines for the Treatment of Infections Caused by Multidrug-Resistant Gram-Negative Bacilli (Endorsed by European Society of Intensive Care Medicine). Clin. Microbiol. Infect..

[8] Tamma P.D., Heil E.L., Justo J.A., Mathers A.J., Satlin M.J., Bonomo R.A. (2024). Infectious Diseases Society of America 2024 Guidance on the Treatment of Antimicrobial-Resistant Gram-Negative Infections. Clin. Infect. Dis..

[9] Aslan A.T., Tanriverdi E.S., Kaya S.Y., Dalgan G., Saltoğlu N., Yılmaz E., Çiçek Y., Yılmaz M., Erol Ç., Azap Ö.K. (2025). Comparison of Ceftazidime-Avibactam with Other Appropriate Antimicrobial Therapy for the Treatment of OXA-48- or KPC-Producing Enterobacterales Infections in Türkiye: A Multi-Centre Retrospective Matched-Cohort Study. Int. J. Antimicrob. Agents.

[10] Tumbarello M., Raffaelli F., Giannella M., Mantengoli E., Mularoni A., Venditti M., De Rosa F.G., Sarmati L., Bassetti M., Brindicci G. (2021). Ceftazidime-Avibactam Use for Klebsiella Pneumoniae Carbapenemase-Producing K. *Pneumoniae* Infections: A Retrospective Observational Multicenter Study. Clin. Infect. Dis..

[11] Wunderink R.G., Giamarellos-Bourboulis E.J., Rahav G., Mathers A.J., Bassetti M., Vazquez J., Cornely O.A., Solomkin J., Bhowmick T., Bishara J. (2018). Effect and Safety of Meropenem-Vaborbactam versus Best-Available Therapy in Patients with Carbapenem-Resistant Enterobacteriaceae Infections: The TANGO II Randomized Clinical Trial. Infect. Dis. Ther..

[12] Gatti M., Cosentino F., Giannella M., Viale P., Pea F. (2024). Clinical Efficacy of Cefiderocol-Based Regimens in Patients with Carbapenem-Resistant *Acinetobacter Baumannii* Infections: A Systematic Review with Meta-Analysis. Int. J. Antimicrob. Agents.

[13] Gatti M., Cosentino F., Giannella M., Viale P., Pea F. (2024). In Reply to the Letter to the Editor Regarding “Clinical Efficacy of Cefiderocol-Based Regimens in Patients with Carbapenem-Resistant *Acinetobacter baumannii* Infections: A Systematic Review with Meta-Analysis”. Int. J. Antimicrob. Agents.

[14] Shields R.K., Abbo L.M., Ackley R., Aitken S.L., Albrecht B., Babiker A., Burgoon R., Cifuentes R., Claeys K.C., Curry B.N. (2025). Effectiveness of Ceftazidime–Avibactam versus Ceftolozane–Tazobactam for Multidrug-Resistant *Pseudomonas aeruginosa* Infections in the USA (CACTUS): A Multicentre, Retrospective, Observational Study. Lancet Infect. Dis..

[15] Hobson C.A., Pierrat G., Tenaillon O., Bonacorsi S., Bercot B., Jaouen E., Jacquier H., Birgy A. (2022). *Klebsiella pneumoniae* Carbapenemase Variants Resistant to Ceftazidime-Avibactam: An Evolutionary Overview. Antimicrob. Agents Chemother..

[16] Ding L., Shen S., Chen J., Tian Z., Shi Q., Han R., Guo Y., Hu F. (2023). *Klebsiella pneumoniae* Carbapenemase Variants: The New Threat to Global Public Health. Clin. Microbiol. Rev..

[17] Abniki R., Tashakor A., Masoudi M., Mansury D. (2024). Global Resistance of Imipenem/Relebactam against Gram-Negative Bacilli: Systematic Review and Meta-Analysis. Curr. Ther. Res..

[18] Di Bella S., Giacobbe D.R., Maraolo A.E., Viaggi V., Luzzati R., Bassetti M., Luzzaro F., Principe L. (2021). Resistance to Ceftazidime/Avibactam in Infections and Colonisations by KPC-Producing Enterobacterales: A Systematic Review of Observational Clinical Studies. J. Glob. Antimicrob. Resist..

[19] Tam V.H., Chang K.-T., Zhou J., Ledesma K.R., Phe K., Gao S., Van Bambeke F., Sánchez-Díaz A.M., Zamorano L., Oliver A. (2017). Determining β-Lactam Exposure Threshold to Suppress Resistance Development in Gram-Negative Bacteria. J. Antimicrob. Chemother..

[20] Sumi C.D., Heffernan A.J., Lipman J., Roberts J.A., Sime F.B. (2019). What Antibiotic Exposures Are Required to Suppress the Emergence of Resistance for Gram-Negative Bacteria? A Systematic Review. Clin. Pharmacokinet..

[21] Gatti M., Cojutti P.G., Pea F. (2024). Impact of Attaining Aggressive vs. Conservative PK/PD Target on the Clinical Efficacy of Beta-Lactams for the Treatment of Gram-Negative Infections in the Critically Ill Patients: A Systematic Review and Meta-Analysis. Crit. Care.

[22] Al-Shaer M.H., Rubido E., Cherabuddi K., Venugopalan V., Klinker K., Peloquin C. (2020). Early Therapeutic Monitoring of β-Lactams and Associated Therapy Outcomes in Critically Ill Patients. J. Antimicrob. Chemother..

[23] Gatti M., Rinaldi M., Cojutti P.G., Bonazzetti C., Siniscalchi A., Tonetti T., Ambretti S., Tedeschi S., Giannella M., Viale P. (2025). A Pre-Post Quasi-Experimental Study of Antimicrobial Stewardship Exploring the Impact of a Multidisciplinary Approach Aimed at Attaining an Aggressive Joint Pharmacokinetic/Pharmacodynamic Target with Ceftazidime/Avibactam on Treatment Outcome of KPC-Producing Klebsiella Pneumoniae Infections and on Ceftazidime/Avibactam Resistance Development. Antimicrob. Agents Chemother..

[24] Gatti M., Pea F. (2023). Jumping into the Future: Overcoming Pharmacokinetic/Pharmacodynamic Hurdles to Optimize the Treatment of Severe Difficult to Treat-Gram-Negative Infections with Novel Beta-Lactams. Expert. Rev. Anti-Infect. Ther..

[25] Gatti M., Pea F. (2021). Continuous versus Intermittent Infusion of Antibiotics in Gram-Negative Multidrug-Resistant Infections. Curr. Opin. Infect. Dis..

[26] Pai Mangalore R., Ashok A., Lee S.J., Romero L., Peel T.N., Udy A.A., Peleg A.Y. (2022). Beta-Lactam Antibiotic Therapeutic Drug Monitoring in Critically Ill Patients: A Systematic Review and Meta-Analysis. Clin. Infect. Dis..

[27] Gatti M., Cojutti P.G., Bartoletti M., Tonetti T., Bianchini A., Ramirez S., Pizzilli G., Ambretti S., Giannella M., Mancini R. (2022). Expert Clinical Pharmacological Advice May Make an Antimicrobial TDM Program for Emerging Candidates More Clinically Useful in Tailoring Therapy of Critically Ill Patients. Crit. Care.

[28] Gatti M., Pea F. (2023). The Expert Clinical Pharmacological Advice Program for Tailoring on Real-Time Antimicrobial Therapies with Emerging TDM Candidates in Special Populations: How the Ugly Duckling Turned into a Swan. Expert. Rev. Clin. Pharmacol..

[29] Gatti M., Rinaldi M., Bonazzetti C., Siniscalchi A., Tonetti T., Ambretti S., Giannella M., Viale P., Pea F. (2025). A Pre-Post Quasi-Experimental Study of the Impact of TDM-Guided Aggressive Pharmacokinetic/Pharmacodynamic Target Attainment of Continuous Infusion Ceftolozane/Tazobactam Monotherapy in Treating Severe *Pseudomonas aeruginosa* Infections: A Strategy Useful for Raising the Bar?. J. Antimicrob. Chemother..

